# Blockade of beta-adrenergic receptors reduces cancer growth and enhances the response to anti-CTLA4 therapy by modulating the tumor microenvironment

**DOI:** 10.1038/s41388-021-02170-0

**Published:** 2022-01-11

**Authors:** Klaire Yixin Fjæstad, Anne Mette Askehøj Rømer, Victor Goitea, Astrid Zedlitz Johansen, Marie-Louise Thorseth, Marco Carretta, Lars Henning Engelholm, Lars Grøntved, Niels Junker, Daniel Hargbøl Madsen

**Affiliations:** 1 National Center for Cancer Immune Therapy (CCIT-DK), Department of Oncology, Copenhagen University Hospital—Herlev and Gentofte, Herlev, Denmark; 2 Department of Immunology and Microbiology, University of Copenhagen, Copenhagen, Denmark; 3 Department of Science and Environment, Roskilde University, Roskilde, Denmark; 4 Department of Biochemistry and Molecular Biology, University of Southern Denmark, Odense, Denmark; 5 Finsen Laboratory, Biotech Research and Innovation Centre, University of Copenhagen, Copenhagen, Denmark; 6 Department of Oncology, Copenhagen University Hospital—Herlev and Gentofte, Herlev, Denmark

**Keywords:** Cancer microenvironment, Tumour angiogenesis, Tumour immunology

## Abstract

The development of immune checkpoint inhibitors (ICI) marks an important breakthrough of cancer therapies in the past years. However, only a limited fraction of patients benefit from such treatments, prompting the search for immune modulating agents that can improve the therapeutic efficacy. The nonselective beta blocker, propranolol, which for decades has been prescribed for the treatment of cardiovascular conditions, has recently been used successfully to treat metastatic angiosarcoma. These results have led to an orphan drug designation by the European Medicines Agency for the treatment of soft tissue sarcomas. The anti-tumor effects of propranolol are suggested to involve the reduction of cancer cell proliferation as well as angiogenesis. Here, we show that oral administration of propranolol delays tumor progression of MCA205 fibrosarcoma model and MC38 colon cancer model and increases the survival rate of tumor bearing mice. Propranolol works by reducing tumor angiogenesis and facilitating an anti-tumoral microenvironment with increased T cell infiltration and reduced infiltration of myeloid-derived suppressor cells (MDSCs). Using T cell deficient mice, we demonstrate that the full anti-tumor effect of propranolol requires the presence of T cells. Flow cytometry-based analysis and RNA sequencing of FACS-sorted cells show that propranolol treatment leads to an upregulation of PD-L1 on tumor associated macrophages (TAMs) and changes in their chemokine expression profile. Lastly, we observe that the co-administration of propranolol significantly enhances the efficacy of anti-CTLA4 therapy. Our results identify propranolol as an immune modulating agent, which can improve immune checkpoint inhibitor therapies in soft tissue sarcoma patients and potentially in other cancers.

## Introduction

Soft tissue sarcoma (STS) is a heterogeneous group of rare mesenchymal tumors that represent approximately 1% of all adult malignancies. The main treatment option for most patients with localized disease is surgical resection, followed by radiotherapy and chemotherapy in selected subtypes [1]. Patients with metastatic diseases have poor clinical outcomes, with an overall survival rate of approximately 12–18 months [2]. Over the past decade, the development of immune checkpoint inhibitors (ICIs), which aim to boost anti-tumor T cell activity, has offered new treatment options for patients with melanoma and epithelial cancer, either as a single agent or in combination with conventional therapies [3]. However, ICIs have achieved suboptimal results on STS patients in two prospective clinical trials (SARC028 and Alliance A091401) of anti-PD1 alone, or in combination with anti-CTLA4 [4, 5]. Current adjuvant treatment to ICI for STS patients, such as axitinib, imposes significant additional toxicity [6]. These studies prompted the combination of ICIs with other less toxic immune modulatory agents to enhance the efficacy.

In recent years, beta-adrenergic signaling has been identified as a contributor to tumorigenesis, tumor progression, and metastasis [7–9]. The sympathetic nervous system maintains basal concentrations of circulating catecholamines, including epinephrine (Epi) and norepinephrine (NE), which signal through adrenergic receptors [10]. The levels of Epi and NE elevate in response to intermittent or chronic stress stimuli, and during tumor progression, neurons from surrounding normal tissues can be recruited to the tumor, further increasing the intratumoral NE level [11, 12]. Beta-adrenergic receptors (ADRBs) are widely expressed in normal tissue, and they are overexpressed in multiple cancer types such as colon, lung, and breast cancer [13]. In the tumor microenvironment (TME), cancer cells, as well as immune cells frequently express ADRBs [14]. Beta blockers are a class of drugs that competitively blocks the ADRBs and thereby reduces beta-adrenergic signaling. Propranolol, a nonselective beta blocker, was first approved in 1967 for cardiovascular indications. However, over the last 50 years, it has been approved for the management of a wide range of other indications, such as essential tremor, prophylaxis of migraines, and benign vascular tumors in infants [15]. Propranolol treatment has shown to be safe in both adult and pediatric cases [16, 17], with few mild side effects, including gastrointestinal disturbances, dizziness, fatigue, and hypotension. The side effects can be effectively managed without the discontinuation of the medication.

Retrospective studies have shown a correlation between beta blocker usage and reduced overall cancer-associated mortality among cancer patients [18]. The combination of propranolol with conventional cancer therapies such as chemotherapy has produced striking responses in hard-to-treat cases such as metastatic angiosarcoma [19–21]. The clinical effectiveness of propranolol against angiosarcoma has resulted in an Orphan Drug Designation by the European Medicines Agency (EMA) for the use against STS. Despite promising results in retrospective studies and small-scale clinical studies, the mechanism of action of propranolol in STS is still not completely understood. In angiosarcoma models of immune deficient mice, propranolol has been shown to reduce cancer cell proliferation and tumor angiogenesis [22]. However, solid tumors consist of not only cancer cells, but also immune cells, forming a complex and dynamic TME [23, 24]. The effect of ADRB stimulation on immune cells in the context of cancer is not well established. While some studies have shown that acute ADRB activation may improve immune function through natural killer (NK) cell mobilization [25], others have observed impaired functions of T cells in the presence of ADRB stimulation [26]. In a preclinical study, ADRB activation increased the generation of myeloid-derived suppressor cells (MDSCs) in vitro, and enhanced their immunosuppressive functions in murine breast cancer models, whereas blockade of ADRB signaling by propranolol reduced the number of MDSCs in both spleen and tumor of tumor bearing mice [27]. In clinical settings, propranolol has been shown to reduce surgically induced elevation in peripheral regulatory T cells (Tregs) in breast cancer patients undergoing radical mastectomy [28].

In this study, we investigated if ADRB signaling could alter the composition of the TME in STS using a syngeneic mouse fibrosarcoma model. We demonstrate that propranolol, as a single agent treatment, reduces tumor angiogenesis and improves the anti-tumor response by increasing the number of tumor infiltrating T cells. In addition, propranolol potently reduces intratumoral MDSCs and alters the gene expression profile of tumor associated macrophages (TAMs). Finally, we show that propranolol enhances the response to anti-CTLA4 treatment. Our data suggest that ADRB signaling contributes to the formation of an immunosuppressive TME in STS and identifies propranolol as an immune modulating agent that could increase the efficacy of checkpoint inhibitor therapy.

## Results

### Blockade of ADRB signaling delays sarcoma growth

**Fig. 1:**
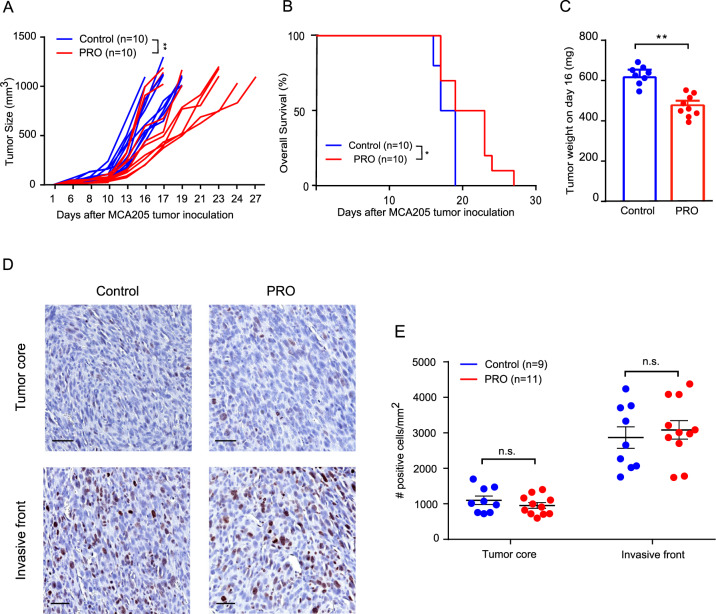
Pharmacological ADRB blockade by propranolol delays tumor growth of MCA205 fibrosarcoma. **A**, **B** Tumor growth curves (**A**) and survival rates (**B**) of control, and propranolol (PRO) treated mice (*n* = 10). **C** Tumor weight on day 16 (*n* = 8–9). **D** Representative Ki67 (brown) IHC staining on MCA205 tumor tissues from control and propranolol treated mice; hematoxylin counterstain; scale bar 50 µm. **E** Dot plot showing quantification of Ki67 staining in nine control mice and 11 propranolol treated mice. **p < 0.01, n.s. not significant, according to multiple *t* test with Bonferroni correction. Tumor growth and survival data were analyzed using TumGrowth software. Mean ± SEM are depicted.

To investigate the importance of ADRB signaling for sarcoma growth, we evaluated the effect of the nonselective ADRB blocker propranolol on a murine model of fibrosarcoma. C57BL/6 mice were inoculated s.c. with MCA205 fibrosarcoma cells and propranolol was administered orally via the drinking water. Propranolol treatment resulted in delayed tumor growth and increased median survival rate of the mice from 18 days to 21 days (Fig. 1A, B). To confirm the anti-tumor effect of propranolol, all tumors were excised and weighed on day 16 in a separate experiment. The weight of tumors from propranolol treated mice were significantly lower than control mice (Fig. 1C). Since propranolol has been reported to have anti-proliferative effects on a variety of epithelium-derived cancer cells [29], we investigated if the delay in tumor growth could be due to a direct inhibition of MCA205 proliferation. We first confirmed the gene expression of *Adrb1* and *Adrb2* in in vitro cultured MCA205 cells by qRT-PCR (Supplementary Fig. S1A). By subjecting cultured MCA205 cancer cells to propranolol in concentrations ranging from 0.4 µM to 200 µM, we observed a dose dependent inhibition of MCA205 proliferation and reduction of viability in vitro (Supplementary Fig. S1B, C). A similar effect was observed on the murine angiosarcoma cell line SVR and murine colon adenocarcinoma cell line MC38 (Supplementary Fig. S1D–G). To examine if an anti-proliferative effect could be observed in vivo, we stained tumor sections from control and propranolol groups for the proliferation marker Ki67 by immunohistochemistry. Surprisingly, the expression levels of Ki67 in tumors from both groups were similar, both in the tumor core and at the invasive front (Fig. 1D, E). This indicated that the effect of propranolol on tumor growth in vivo was unlikely due to direct inhibition of cancer cell proliferation.

**Fig. 2:**
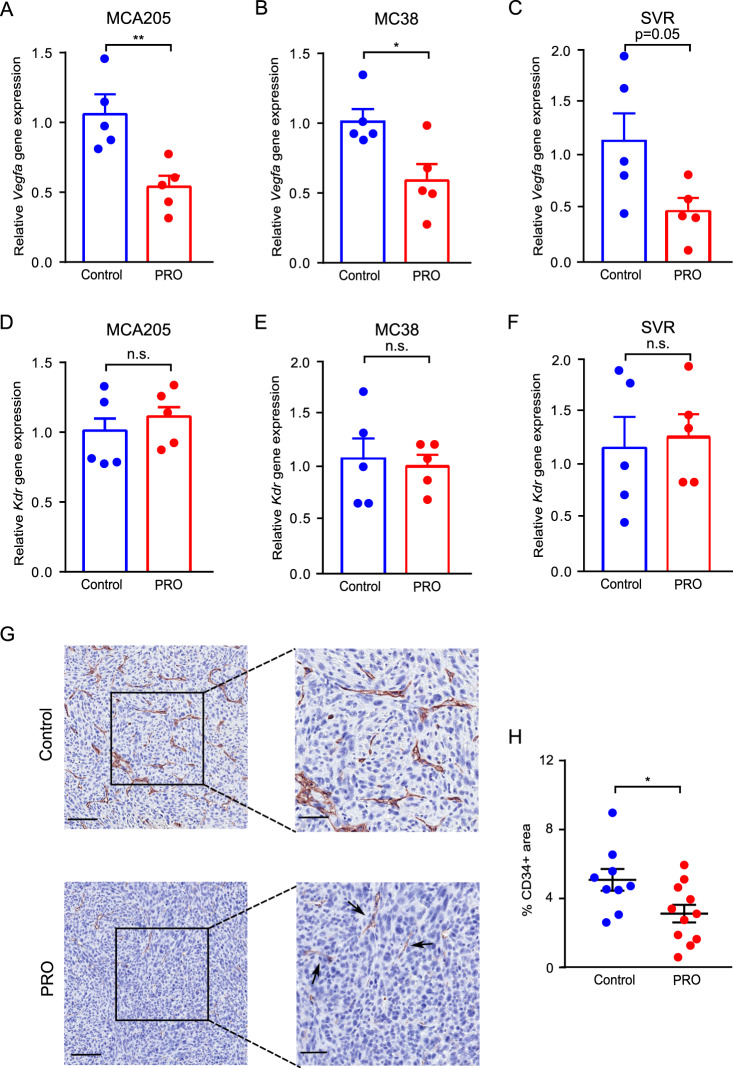
Propranolol treatment reduces tumor angiogenesis. **A**–**C** Quantification of angiogenic marker *Vegfa* gene expression in MCA205 (**A**), MC38 (**B**), and SVR (**C**) tumors from control mice and propranolol (PRO) treated mice by qRT-PCR (*n* = 5). **D**–**F** Quantification of *Kdr* gene expression in MCA205 (**D**), MC38 (**E**), and SVR (**F**) tumors from control mice and propranolol (PRO) treated mice by qRT-PCR (*n* = 5). **G** Representative CD34 (brown) IHC staining of MCA205 tumor tissue from control mice or propranolol treated mice; hematoxylin counterstain; scale bar 100 µm (left panels), and 50 µm (right panels). **H** Dot plot showing quantification of CD34 staining by percentages of DAB + area in 9 control mice and 11 propranolol treated mice. *p < 0.05, **p < 0.01, n.s. Not significant, according to multiple *t* test with Bonferroni correction. Mean ± SEM are depicted.

Propranolol has also been demonstrated to be a potent anti-angiogenic agent in preclinical studies by reducing the expression of vascular endothelial growth factor A (VEGFA) [16]. Therefore, we measured *Vegfa* gene expression in MCA205 tumor RNA from control and propranolol treated mice using qRT-PCR. Propranolol treatment resulted in a 50% reduction in intratumoral *Vegfa* gene expression (Fig. 2A). Similarly, we observed a reduction of *Vegfa* gene expression in RNA samples from propranolol treated MC38 tumors (Fig. 2B), and a trend toward a decrease in propranolol treated SVR tumors (Fig. 2C). The expression levels of the *Kdr* gene encoding vascular endothelial growth factor receptor 2 (VEGFR2), the primary receptor of VEGFA, were similar between control and propranolol treated groups in all three tumor models tested (Fig. 2D–F). To confirm the anti-angiogenic effect of propranolol, we immuno-stained paraffin embedded tissue sections of MCA205 tumors for the endothelial marker CD34 and quantified the CD34-positive area in the tumor core, excluding necrotic areas. Tumor sections from the propranolol treated group had a significantly lower CD34-positive area compared to the control group, confirming the anti-angiogenic effect of propranolol (Fig. 2G, H). We did not observe any changes in the gene expression of lymphatic vessel marker *Lyve1* in tumor RNA upon propranolol treatment in any of the three models tested (Supplementary Fig. S2A–C). This indicated that lymphatic vessel growth was not affected by propranolol.

### The anti-tumor effect of propranolol involves T cell activity

**Fig. 3:**
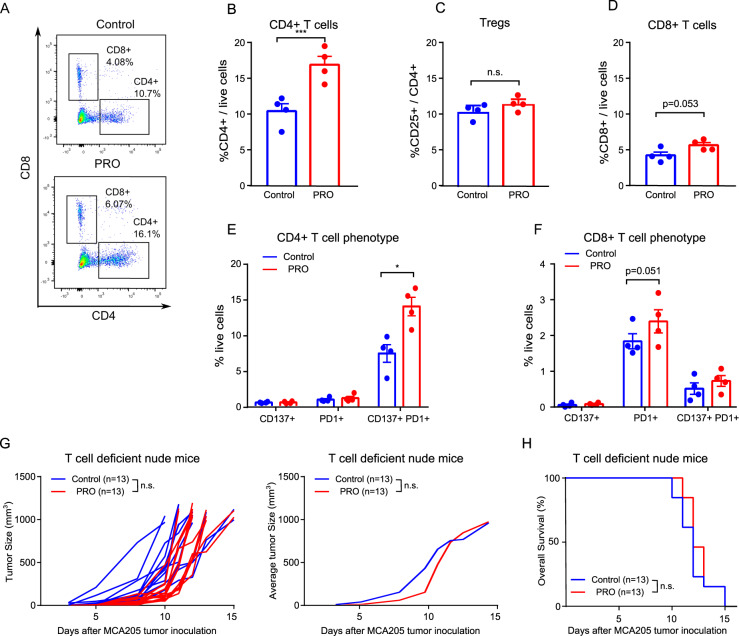
Propranolol treatment increases CD4 + T cell infiltration in MCA205 tumors. **A**–**F** Single cell suspensions were made from excised MCA205 tumors at the experimental endpoint and analyzed by flow cytometry. **A** Representative flow cytometry dot plot of CD4 + and CD8 + T cells in the TME. Quantification of CD4 + T cells (**B**), regulatory T cells (**C**), and CD8 + T cells (**D**) (*n* = 4). Quantification of CD137 + , PD1 + CD137 + PD1 + CD4 + T cells (**E**), and CD8 + T cells (**F**) among all live cells in the TME (*n* = 4). **G**, **H** Tumor growth kinetics (**E**) and survival rates (**F**) of control, and propranolol treated (PRO) nude mice (*n* = 13). For tumor growth and Kaplan–Meier curves, statistical analyses were performed using TumGrowth software. For other comparisons, multiple *t* tests with Bonferroni correction for multiple comparison were used. ***p < 0.005, n.s. Not significant. Mean ± SEM are depicted.

To investigate if the mechanisms of action of propranolol extended beyond anti-angiogenic effects, we analyzed if propranolol affected the infiltration of T cells. The cellular composition of the lymphoid compartment of the MCA205 tumors was analyzed by multicolor flow cytometry. Propranolol led to an increased infiltration of CD4 + T cells into the tumors, without altering the ratio of Tregs (CD25 + CD4+) to total CD4 + T cells (Fig. 3A–C). There was also a trend toward an increase in intratumoral CD8 + T cells in the propranolol treated mice (Fig. 3D). By analyzing the expression of T cell activation marker CD137 and exhaustion marker PD1 on CD4 + and CD8 + T cells, we observed an increase in CD137 + PD1 + CD4 + T cells, and a trend towards increase in PD1 + CD8 + T cells in the TME (Fig. 3E, F). We did not observe changes in the abundance of NK cells in the tumors (Supplementary Fig. S2E).

To directly test if the therapeutic efficacy of propranolol is dependent on the presence of T cells, we inoculated T cell deficient nude mice with MCA205 cells and treated them with propranolol. In nude mice, the growth rate of MCA205 tumors and survival rate of mice were comparable between control and propranolol treated mice (Fig. 3G, H). An experiment with immune competent mice was conducted using the same cell line stock concurrently, which confirmed the anti-tumor effect of propranolol in immune competent mice (data not shown). These results indicated that the anti-tumor effect of propranolol depends on the presence of T cells.

### Blockade of ADRB signaling affects the myeloid compartment of the TME

**Fig. 4:**
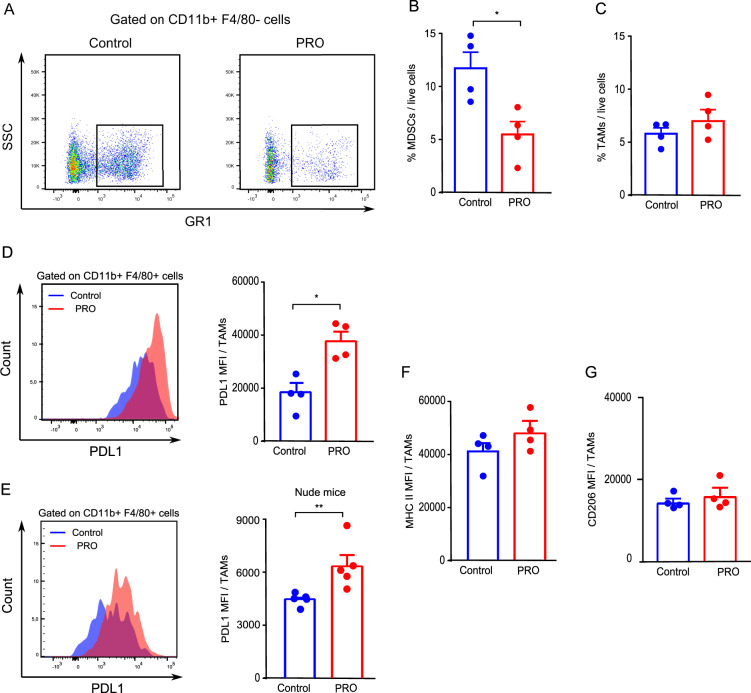
ADRB blockade by propranolol reduces intratumoral MDSCs and upregulates PDL1 expression on tumor associated macrophages (TAMs). Single cell suspensions were made from excised MCA205 tumors in immune competent mice and T cell deficient nude mice at the experimental endpoint and analyzed by flow cytometry (*n* = 3–5). **A**, **B** Representative flow cytometry dot plot (**A**) and quantification of MDSCs (CD11b + F4/80- GR1 + ) (**B**) in the TME of tumors from control and propranolol (PRO) groups in immune competent mice. **C** Quantification of TAMs (CD11b + F4/80+) in samples from control and propranolol treated immune competent mice. **D**, **E**, Representative histograms and quantifications of mean fluorescence intensity (MFI) of PDL1 on TAMs from immune competent mice (**D**), and T cell deficient nude mice (**E**). MFI quantification of MHC II (**F**) and CD206 (**G**) expression on TAMs in immune competent mice. Multiple *t* tests with Bonferroni correction for multiple comparison were used for statistical testing. *p < 0.05, **p < 0.01. Mean ± SEM are depicted.

Since immunosuppressive cells of the myeloid lineage can contribute to tumor progression in a variety of cancer types and influence the recruitment and function of T cells [30], we investigated if propranolol affected the cellular composition of the myeloid compartment in the TME of MCA205 tumors. Flow cytometry-based analysis of the TME showed that propranolol treatment led to a striking reduction in the abundance of intratumoral myeloid-derived suppressor cells (MDSCs) (Fig. 4A, B). No difference was observed in the numbers of tumor associated macrophages (TAMs) (Fig. 4C). We next examined the phenotype of MDSCs and TAMs by measuring the surface expression of MHC II and PDL1 by flow cytometry. No difference was observed between the expression of these molecules in MDSCs (Supplementary Fig. S2G). However, interestingly, we found an increase in PDL1 expression (Fig. 4D), and a trend towards increased MHC II expression on TAMs from propranolol treated mice (Fig. 4F). There was no difference in the expression level of the M2 marker CD206 (Fig. 4G). In nude mice, we also observed an increase in PDL1 expression on TAMs in MCA205 tumors from propranolol treated mice (Fig. 4E), although less pronounced than in the presence of T cells (compare Fig. 4D, E). Although PDL1 expression on tumor cells is associated with poor prognosis in patients, a recent study indicates that PDL1 expression on macrophages is associated with a “hot” tumor phenotype and increased T cell infiltration [31]. In summary, our data suggest that macrophages can be affected directly by the blockade of ADRB.

### ADRB signaling affects the phenotype of macrophages in vitro

**Fig. 5:**
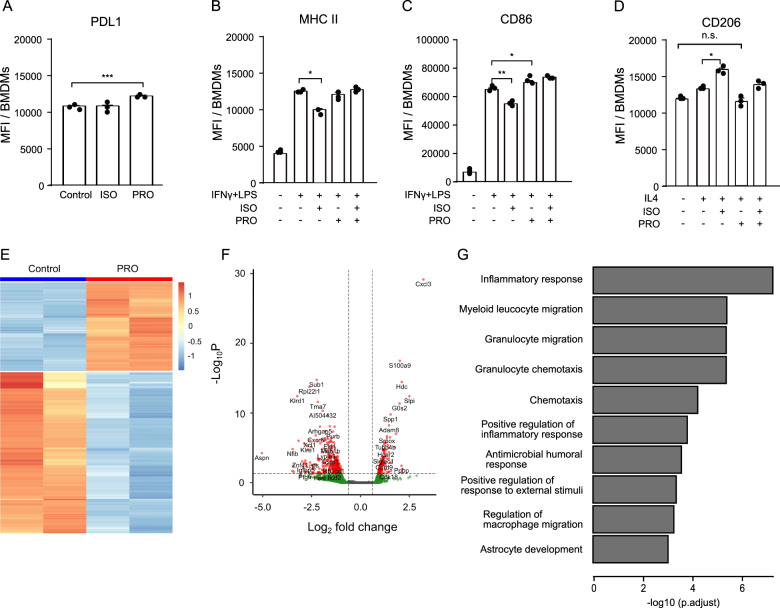
Macrophages are sensitive to ADRB stimulation and propranolol treatment leads to TAMs with distinct gene expression profiles. **A** Quantification of PDL1 MFI on non-polarized macrophages upon isoprenaline (ISO) or propranolol (PRO) treatment. isoprenaline and/or propranolol’s effect on the surface expression of MHC II (**B**), CD86 (**C**), or CD206 (**D**) under M1 polarizing condition with IFNγ and LPS or under M2 polarizing condition with IL4. **E**, **F**, RNA-seq analysis showing heatmap (**E**) and volcano plot (**F**) of genes differentially regulated in TAMs from control or propranolol treated mice. Data show two biological replicates. **G** Gene ontology analysis showing the biological processes most significantly enriched within genes that are differentially expressed between TAMs isolated from control mice or propranolol treated mice. *p < 0.05, **p < 0.01, ***p < 0.005, according to multiple *t* test with Bonferroni correction for multiple comparison. Mean ± SD are depicted.

To test how macrophages respond to ADRB stimulation, we generated BMDMs. The differentiated macrophages were treated with the pan-ADRB agonist isoprenaline or with propranolol. The expression of PDL1 was increased by propranolol treatment while isoprenaline had no effect (Fig. 5A). We then evaluated the expression of the M1 markers CD86 and MHC II, and the M2 marker CD206 upon ADRB stimulation or subsequent blockade under M1 or M2 polarizing conditions. Isoprenaline dampened BMDMs’ response to IFNγ and LPS stimulation, demonstrated by a reduction in MHC II and CD86 expression. Subsequent blocking of ADRB with propranolol rescued the expression of these two markers (Fig. 5B, C). In the presence of the M2 polarizing cytokine IL4, isoprenaline enhanced the expression of CD206, skewing BMDMs further towards M2-like polarization. Blockade of ADRB by propranolol neutralized the effect (Fig. 5D). Interestingly, propranolol alone dampened the M2 polarizing effect of IL4; the expression levels of MHC II, CD86, PDL1, and CD206 on IL4 and propranolol treated BMDMs were similar to those of untreated control cells (Fig. 5D, and Supplementary Fig. S3A–C).

### TAMs from MCA205 tumors of propranolol treated mice have a distinct gene expression profile

Ex vivo generated BMDMs are responsive to ADRB signaling and blockade. To examine how propranolol affects the phenotype of TAMs in vivo, we FACS-sorted TAMs from MCA205 tumors of propranolol treated or control mice and extracted RNA for RNA sequencing (RNAseq). TAMs from propranolol treated mice had a distinct gene expression profile compared to TAMs from control mice (Fig. 5E). We identified 390 genes, which were regulated by more than 1.5-fold (*p* < 0.05) in response to propranolol treatment (Fig. 5F). The top ten most upregulated and down regulated genes are listed in Supplementary Table 3, and the complete list of differentially regulated genes can be found in Supplementary Table 4. Gene ontology enrichment analysis revealed that the differentially regulated genes were involved in biological processes, including inflammatory response, myeloid leukocyte migration, and granulocyte migration (Fig. 5G).

### Propranolol enhances the effect of anti-CTLA4 treatment

**Fig. 6:**
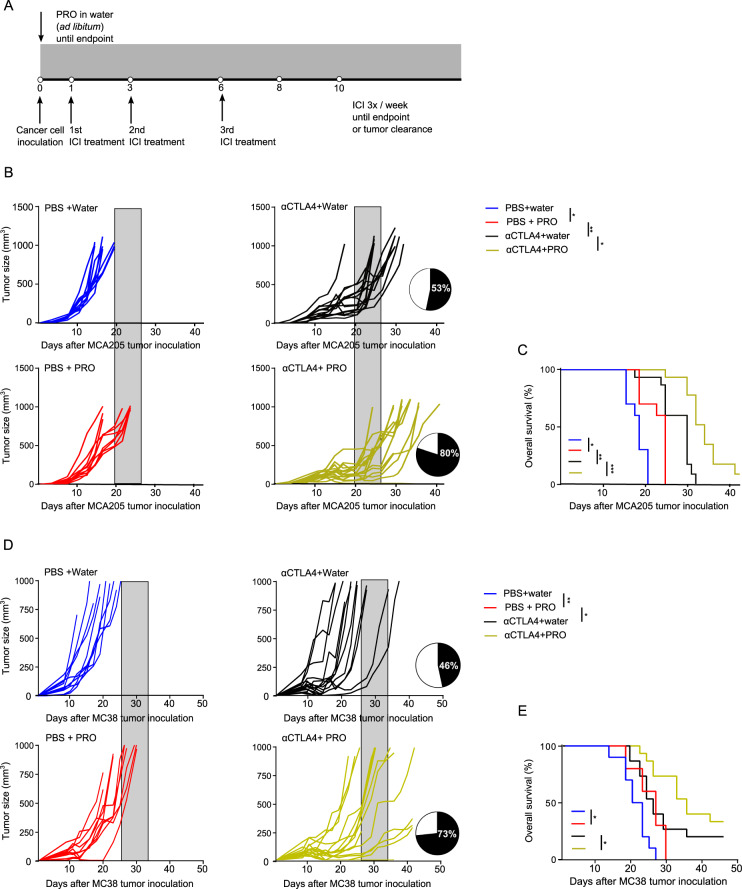
ADRB blockade by propranolol (PRO) improves the efficacy of anti-CTLA4 in MCA205 and MC38 tumor model. **A** Treatment regimen and experimental setup of immune checkpoint inhibitor (ICI) tumor studies. **B**, **C** Tumor growth kinetics (**B**) and Kaplan–Meier survival curves (**C**) of C57BL/6 mice inoculated with MCA205 cancer cells, treated with anti-CTLA4 and PRO. **D**, **E** Tumor growth kinetics (**D**) and Kaplan–Meier survival curves (**E**) of C57BL/6 mice inoculated with MC38 cancer cells, treated with anti-CTLA4 and PRO. Each line represents one animal. *n* = 10–15 per group. Pie charts indicate the response rate of treated mice. Therapeutic response is defined as having stable tumor volume for more than 7 days (four measurements). Shaded areas, included for easy comparison of the different treatment groups, represent the terminal tumor growth time frame of the PBS + water group. Statistical analyses were performed using TumGrowth software. *p < 0.05, **p < 0.01, ***p < 0.005.

**Fig. 7:**
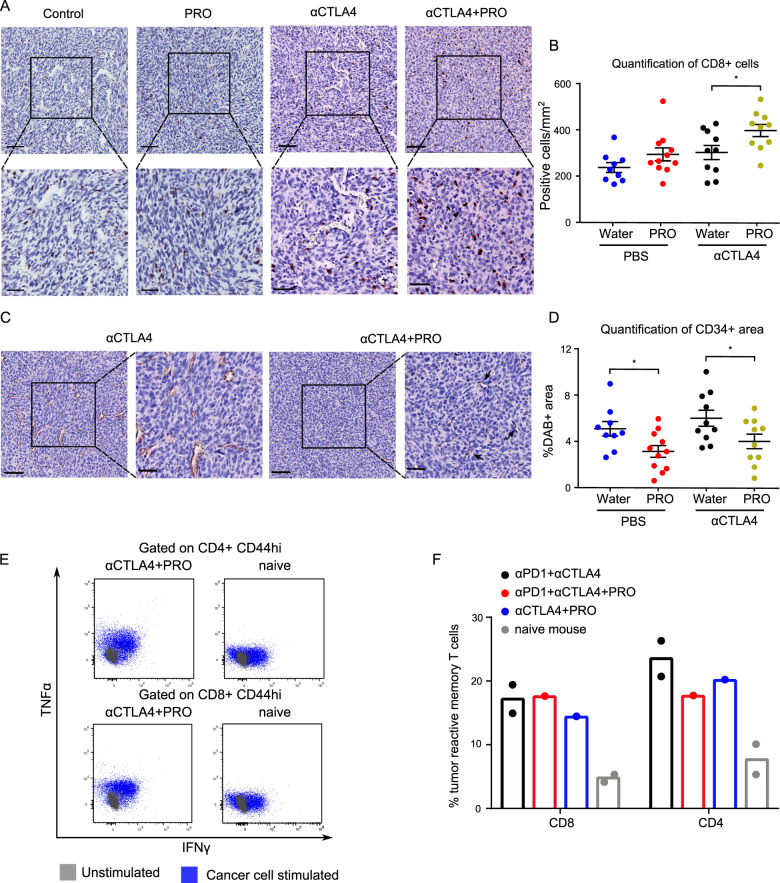
Propranolol combined with anti-CTLA4 increases the number of intratumoral CD8 + T cells, reduces tumor angiogenesis, and provides long lasting immune memory against MCA205 cancer cells. **A**–**C** Representative CD8 (**A**) and CD34 (**C**) IHC staining of MCA205 tumor tissues; hematoxylin counterstain; scale bar 100 µm (upper panels), and 50 µm (lower panels). **B**, **D** Dot plot showing quantification of CD8 (**B**), and CD34 staining (**D**). Splenocytes from cured mice were re-stimulated ex vivo with MCA205 cancer cells for 24 h, and intracellular cytokine expressions were quantified by flow cytometry. **E** Representative flow cytometry dot plots showing the expression of TNFα and IFNγ on CD4 + T cells (upper panels) or CD8 + T cells (lower panels) with memory phenotype from naïve mice (left panels) or anti-CTLA4 + PRO treated mice that had shown complete tumor regression(right panels). **F** Percentages of tumor reactive T cells (IFNy + or TNFa + ) with memory phenotype (CD44hi) among CD8 + or CD4 + T cells after 24 h ex vivo re-stimulation. *p < 0.05 according to multiple *t* test with Bonferroni correction. Mean ± SEM are depicted.

A high abundance of tumor infiltrating T cells is a predictive marker of ICI efficacy, and a low number of MDSCs has been shown to correlate with an increased chance of responding to anti-CTLA4 therapy [32, 33]. Propranolol treatment led to increased T cell infiltration and reduced intratumoral MDSCs in MCA205 tumors and we, therefore, decided to combine propranolol with ICI therapy (Fig. 6A). First, propranolol was combined with anti-PDL1 treatment. MCA205 tumors were minimally responsive to anti-PDL1 treatment, and the tumor growth and survival rate of mice were comparable between the two ICI treated groups with or without propranolol (Supplementary Fig. S4A, B). Similar results were obtained when propranolol was combined with anti-PD1 treatment (Supplementary Fig. S4C–D), suggesting that ADRB blockade did not affect therapy targeting the PD1/L1 axis in this tumor model. This result indicates that propranolol induced upregulation of PDL1 is not sufficient to make the MCA205 tumors responsive to anti-PD-1/PDL1 therapy. Next, we tested the effect of anti-CTLA4 in combination with propranolol. Anti-CTLA4 alone led to temporary stabilization of MCA205 tumors, before the tumors became resistant to therapy. Interestingly, the addition of propranolol further delayed tumor growth and improved the survival rate compared to anti-CTLA4 alone (Fig. 6B, C). The treatment combination also improved the response rate to treatment, from 8/15 (53%) in anti-CTLA4 group to 12/15 (80%) in anti-CTLA4 + PRO group. One mouse in the anti-CTLA4 + PRO group showed complete tumor clearance, while none in the anti-CTLA4 single treated group achieved tumor free status. To investigate if the anti-tumor effects of propranolol and its ability to enhance the efficacy of anti-CTLA4 therapy also applied to other cancer types, we performed a similar experiment using the MC38 colon cancer model. We observed a similar anti-tumor effect of propranolol as a single agent as well as increased therapeutic efficacy when combined with anti-CTLA4 therapy (Fig. 6D, E). To gain insight into the mechanism of action of the observed synergistic effect, we performed immunohistochemical staining of CD8 and CD34 on paraffin embedded MCA205 tumor tissue sections from mice treated with anti-CTLA4 with or without propranolol. Anti-CTLA4 treated mice had a trend towards increase in tumor infiltrating CD8 + T cells compared to control mice, and the combination of propranolol and anti-CTLA4 led to a further increase in the number of tumor infiltrating CD8 + T cells (Fig. 7A, B). The anti-angiogenic effect of propranolol as a single agent treatment was also observed when used in combination with anti-CTLA4 in both the MCA205 (Fig. 7C, D) and MC38 tumor model (Supplementary Fig. S5A, B).

We then tested if propranolol could increase the response rate of an already very effective treatment regimen of combined anti-PD1 and anti-CTLA4 antibodies. Propranolol did not alter the effectiveness of the maximum dose of ICI treatment (Supplementary Fig. S4E, F). In the ICI treated groups with or without propranolol, 13/15 mice responded to treatment, and three mice experienced complete tumor regression. Out of the six tumor free mice from both ICI treated groups, three mice relapsed after treatment discontinuation.

### Anti-CTLA4 and propranolol treatment can lead to the development of immune memory against MCA205 cancer cells

As described above, complete tumor clearance was observed in a limited number of mice after combination treatment of ICI and PRO. To investigate if these mice developed immune memory against the cancer cells, we re-inoculated the mice with MCA205 cancer cells three months after tumor clearance. All mice remained tumor free during the one-month observation period, while five out of five tumor naïve mice developed tumors within 6 days of inoculation. To evaluate if cured mice developed long lasting T cell memory, isolated splenocytes from tumor free mice were stimulated with MCA205 cells and the expression levels of IFNγ and TNFα in T cells were examined by flow cytometry. T cells positive for either IFNγ or TNFα were defined as tumor reactive. CD44 was used to identify murine memory T cells. Splenocytes from tumor free mice treated with anti-PD1 + anti-CTLA4, anti-PD1 + anti-CTLA4 + PRO, and anti-CTLA4 + PRO harbored higher number of tumor reactive T cells with a memory phenotype against MCA205 cancer cells, compared to splenocytes from tumor naïve mice (Fig. 7E, F). The response levels between all tumor free mice were similar. The data supports that propranolol exerts anti-tumor effects that involve the formation of an anti-tumoral immune environment, and it suggests that combinations of propranolol and ICI could lead to durable therapeutic responses.

## Discussion

Clinical trials of checkpoint inhibitors have achieved suboptimal results in STS patients [4]; therefore, there is a need to improve immunological responses towards the tumor with immune modulatory agents. Retrospective analyses have shown a correlation between post-diagnostic use of nonselective beta blockers and increased relapse-free survival in a variety of cancer types [34, 35]. In this study, we demonstrated the potential of propranolol as a potent immune modulatory agent that can enhance the therapeutic efficacy of anti-CTLA4 checkpoint inhibitor therapy in STS using a murine fibrosarcoma model. We also show a similar effect of propranolol in a murine model of colon cancer.

Preclinical studies on the therapeutic effect of propranolol have identified direct anti-proliferative effect on angiosarcoma cells using in vitro assays and immune deficient mice. In our study, we did not observe a difference in Ki67 expression in tumor sections, indicating that a direct anti-proliferative effect of propranolol is not a major contributing factor to its anti-tumor effects in vivo. Activation of the ADRBs on immune cells has been shown to dampen the inflammatory response towards infectious disease [36, 37]. Here, we show that propranolol treatment resulted in an increased number of tumor infiltrating T cells, with a more prominent effect on CD4 + T cells. The propranolol induced increase in T cell infiltration is essential for the improved tumor control, since T cell deficient nude mice did not benefit from propranolol in terms of tumor growth and survival rate. In immune competent mice, we observed an increase in CD137 + PD1 + activated CD4 + T cells, which suggests that the potentiating effect of propranolol could operate through the increase in T cell recruitment and activation.

The myeloid cell of the TME can inhibit T cell functions, shape the angiogenic network in cancer, and regulate the recruitment of lymphocytes to the tumor [30, 38]. In this study, we observed a striking reduction of MDSCs in the tumor. MDSCs can stimulate angiogenesis and propranolol’s potent anti-angiogenic effect shown in this study could therefore be partially mediated by the reduction of MDSCs in the tumor. Another immune suppressive myeloid cell type, TAMs, are shown to be sensitive to ADBR signaling [39]. Here, we investigated the direct impact of propranolol on macrophage function in vitro using BMDMs. In line with the literature, we observed significant M2 polarizing effect of the ADRB agonist isoprenaline, while propranolol rescued the agonist induced downregulation of the M1-associated markers MHC II and CD86. Interestingly, we observed that propranolol alone was able to neutralize the M2 polarizing effect, which could be related to its ability to inhibit lipin-1, an enzyme suggested to be essential for IL4-mediated macrophage polarization in vitro. [40, 41]. RNA sequencing of FACS-sorted TAMs confirmed that propranolol induced a distinct gene expression profile, and an altered chemokine profile (Supplementary Fig. S3D). These findings suggest that the propranolol induced changes in the lymphoid compartment could be partly caused by an altered TAM-mediated recruitment of lymphocytes.

Propranolol treatment induces a shift of the TME towards a pro-inflammatory state, which is potentially synergistic with ICI therapies. A high number of tumor infiltrating T cells has been associated with increased overall survival and response to immunotherapy in a variety of cancer types, including colorectal cancers, ovarian cancers, and melanoma [42, 43]. Moreover, a low number of MDSCs is associated with improved clinical response towards anti-CTLA4 and anti-PD1/PD-L1 checkpoint inhibitor therapies [44, 45]. By combining propranolol treatment with various ICI antibodies, we demonstrated that ADRB blockade significantly improves the efficacy of anti-CTLA4 therapy. The combination resulted in a reduced tumor growth rate and increased overall survival. In one case, the combination treatment resulted in complete MCA205 tumor clearance. Notably, the tumor free mouse is resistant to subsequent MCA205 tumor re-challenge three months after tumor clearance, highlighting that a durable anti-tumor response was obtained. Analysis of the presence of tumor reactive splenocytes showed that the complete response was accompanied by acquired long lasting T cell memory. This acquired immunity was comparable to that achieved with combined anti-CTLA4 and anti-PD1 treatment.

Few published studies have investigated the effect of ADRB blockade on the efficacy of ICIs. A retrospective analysis of melanoma patients treated with immune therapies showed that the concurrent usage of propranolol correlates with prolonged overall survival [46]. Recently, a phase I clinical trial investigating the combination of propranolol and pembrolizumab in a limited number of patients indicated that the treatment combination is tolerable and could lead to increased response rates [47]. Additionally, a preclinical study has shown that ADRB blockade by propranolol or genetic ablation of *Adrb2* improved anti-PD1 treatment outcome in a murine breast cancer model [48]. In our study, propranolol treatment did not improve the response of MCA205 model to anti-PD1 or anti-PDL1 treatment, which could be due to the difference in cancer types. Further effort needs to be directed to identify the cancer types that can benefit from the combination treatment, and elucidate which ADRB receptor is more critical to the anti-tumor effect of propranolol.

In the context of therapeutic cancer vaccines, propranolol has been demonstrated to improve naïve T cell priming, thereby boosting vaccine efficacy [49]. In another study, anti-CTLA4 enhanced the treatment outcome of a therapeutic cancer vaccine through the improvement of T cell priming in the lymphoid tissues [50]. Therefore, it is possible that propranolol and anti-CTLA4 synergistically stimulate the initial priming phase of an anti-cancer immune response, making this combination treatment particularly effective.

The data presented in this study provide new insights into the therapeutic effect of propranolol on STS and shows that propranolol modulates immune responses against STS by increasing T cell recruitment and by reducing the number of tumor infiltrating MDSCs. The study supports the idea that adrenergic signaling contributes to therapy resistance and strengthens the rationale of combining propranolol, a low cost, and relatively non-toxic adjuvant therapy, with ICI therapies for treating cancer patients.

## Methods

### Mouse studies

Animal experiments were conducted at the animal facility of the Department of Oncology, Herlev Hospital, Denmark, under license issued by the Animal Experiments Inspectorate. Daily maintenance of C57BL/6 and NMRI-nu/nu mouse stocks were performed by the animal caretakers at the animal facility. Experimental mice were females between 8 and 16 weeks of age. The right flank of mice was subcutaneously (s.c.) injected with 1 × 10^6^ MCA205 or 1 × 10^6^ SVR cells or 0.5 × 10^6^ MC38 cells. The mice were randomized into control or treatment groups after cancer cell inoculation. Tumor dimensions (length and width) were measured three times a week with a digital caliper by a blinded researcher. Tumor volumes were calculated by the formula: 0.5 x length x width^2^. The experimental endpoint was defined as tumor volume reaching 1000 mm^3^. Mice were sacrificed by cervical dislocation and the tumors were collected for further analysis unless otherwise noted. Tumors with ulceration were excluded from the analysis.

### Cell lines

The MCA205 cell line was purchased from Merck (SCC173) and cultured in complete growth medium, consisting of RPMI-1640 GlutaMAX™ Supplement HEPES, 20% FBS, 1% sodium pyruvate, 1% nonessential amino acids, and 1% P/S (All from Gibco). The complete growth medium was supplemented with β-mercaptoethanol (Gibco) at a concentration of 55 µM before use. The SVR cell line was purchased from ATCC (CRL-2280) and cultured in complete growth medium, consisting of DMEM, 10% FBS, and 1% P/S (All from Gibco). The MC38 cell line was retrieved from cell line biobank in the group and cultured in complete growth medium, consisting of DMEM, 10% FBS, and 1% P/S (All from Gibco). Cells lines were not tested for mycoplasm contaminations.

### Therapies

(±) Propranolol hydrochloride (propranolol) (Sigma) was dissolved in room temperature drinking water at 0.5 g/L and offered to mice ad libitum, starting on the day of cancer cell inoculation. Water bottles for all mice were changed every 3–4 days. In checkpoint inhibitor treated groups, mice were treated intraperitoneally (i.p.) three times a week, starting 1 day after cancer cell inoculation, with anti-PDL1 mAb (200 μg/mouse; clone 10 F.9G2, BioXcell), anti-CTLA4 mAb (200 μg/mouse; clone 9D9, BioXcell), anti-PD1 mAb (200 μg/mouse; clone RMP1-14, BioXcell), alone or in combinations until endpoint or one week after tumor clearance. Control mice were treated with PBS following the same treatment scheme.

### Bone marrow derived macrophages (BMDMs)

BMDMs were generated from the marrow of femurs and tibias from female C57BL/6 mice. Bones were rinsed in 70% ethanol and in PBS, and the bone marrow cells were flushed with PBS using a 25 G needle. After red blood cell lysis with RBC lysis buffer (Qiagen), cells were cultured in BMDM growth media, consisting of Iscove’s modified Dulbecco medium (IMDM, Sigma Aldrich), supplemented with 20% FBS, 1% P/S, and 20 ng/mL recombinant human M-CSF (R and D system), at a concentration of 1 × 10^6^ cells/mL. The growth medium was refreshed on day 3. Differentiated macrophages were collected on day 7, and the purity determined to be more than 98% by multicolor flow cytometry, using cell surface markers CD11b and F4/80. The macrophages were cultured under different conditions for 24 h for specific macrophage polarization. For M1 polarization of macrophages, 100 ng/mL LPS (Invitrogen) and 40 ng/mL IFNγ (PeproTech) were used. For M2 polarizations, 40 ng/mL IL4 (PeproTech) was used. For adrenergic stimulation and blockade, (±) isoprenaline hydrochloride (isoprenaline) (Sigma Aldrich) or propranolol was dissolved in BMDM growth media and added to the cells.

### Flow cytometry analyses

Excised tumors were minced with scissors and enzymatically digested in RPMI medium containing 2.1 mg/ml collagenase type 1 (Worthington), 75 μg/ml DNase I (Worthington), 5 mM CaCl_2_, and 1% P/S. Cells were then filtered through a 70 μm cell strainer. After red blood cell lysis, cells were incubated with FcR block (Miltenyi) for 10 min at 4 °C. Dead cells were excluded using Zombie Aqua viability dye (BioLegend). For membrane staining, cells were incubated with antibody mix at 4 °C in the dark for 20 min.

For intracellular IFNγ and TNFα staining, isolated splenocytes from tumor free mice were first stimulated with in vitro cultured MCA205 cancer cells in a 24-well plate for 24 h in MCA205 complete growth media. A ratio of 2 × 10^6^ splenocytes to 200,000 cancer cells were used. GolgiPlug (1/1000, BD Biosciences) was added to the mixed culture and incubated for an additional 4 h. Non-adherent cells were collected and stained for T cell markers and CD44 as a marker of murine memory T cells, before permeabilization using a FoxP3/transcription factor staining buffer set (eBioscience), according to manufacturer’s instruction. The cells were stained with anti-IFNγ and anti-TNFα antibodies (Biolegend) at 4 °C dark for 35 min. All antibodies used are listed in Supplementary Table 1. Data were acquired on a FACSCanto II (BD) or Quanteon (NovoCyte) flow cytometer after appropriate compensation using single-stained cells or compensation beads (BD Biosciences). Data were analyzed with Flow Jo software (Tree Star).

### Gene expression analyses

Excised MCA205 tumors were stored in RNAlater (Invitrogen) at −80 °C until RNA extraction. Tumors were placed in buffer RLT (Qiagen), and mechanically homogenized with Tissue Lyser (Qiagen). Total RNA extraction was performed with the RNeasy Mini kit (Qiagen), following the manufacturer’s instruction. A maximum of 1 μg of RNA, measured by NanoDrop Spectrophotometer (Thermo Scientific), was reverse transcribed into cDNA using iScript cDNA synthesis kit (Biorad), according to manufacturer’s protocol. qRT-PCR was performed using the Brilliant III Ultra-Fast SYBR® dye system (Agilent) with ROX as a reference dye. The loaded plates were run on an AriaMX Real-Time PCR System with the thermal profile: 1 cycle at 95 °C for 3 min, followed by 40 cycles of 95 °C for 5 s, 60 °C for 20 s. This was followed by a melting curve analysis of 65–95 °C with 0.5 °C increment, 5 s per step. Quantitative qRT-PCR data were normalized to the expression level of the housekeeping gene *Actb*. Data were analyzed using the 2^–ΔΔCt^ method, and fold change of treated group compared to the control group was calculated. Primer sequences are listed in supplementary table 2.

### Cell sorting and RNA sequencing

For TAM isolation, single cell suspensions of MCA205 tumors from control and propranolol treated mice were labeled with CD45 microbeads (Miltenyi) and the CD45 + cells were purified using magnetic separation. The CD45 + cell fractions were stained with Zombie Aqua viability dye, anti-CD11b, and anti-F4/80 antibodies. CD11b + F4/80+ live cells were sorted by FACS on the ARIA III (BD Biosciences). The purity of samples was tested to be at least 96%. RNA from sorted TAMs were extracted immediately after sorting, using RNeasy Mini Kit (Qiagen) according to the manufacturer’s instructions. The quality of RNA samples was determined by Bioanalyzer (Agilent). Only samples with RNA integrity number (RIN) of more than 7.5 were used for RNA sequencing.

### RNA sequencing

In total 400 ng RNA was prepared for sequencing using polydT enrichment according to the manufacturer’s instructions (Illumina). Library preparation was performed using the NEBNext RNA library prep kit for Illumina. The library quality was assessed using a Fragment Analyzer followed by library quantification using the Illumina library quantification kit. Libraries were sequenced on a NovaSeq 6000 platform (Illumina) to a minimum depth of 30 million reads per sample. Sequenced reads were aligned to the reference mm10 genome for mouse using STAR, version 2.7.1. High-quality reads were filtered by MAPQ scores (MAPQ > 30) using SAMtools. The gene expression count matrix was generated using featureCounts version 1.6.4 and GENCODE gene annotation with the parameters “-p -t exon”. RNAseq data can be accessed on GEO repository (GSE174645).

For all analyses of the RNA seq data R version 4.0.3 was used. Analysis of differentially expressed genes was performed using DESeq2 package version 1.30 (Cut off p-adjusted value <0.05 and log2 fold change >1.5). For the GoSeq analysis, compareCluster from the clusterProfiler package version 3.18.0 was used. Redundant GOterms were excluded using the Simplify command. The Benjamini-Hochberg method and p-adjusted-value cut-off <0.05 were set as criterion for the GOSeq analysis. The ggplot2 package version 3.3.3, was used to visualize the top ten GOterms.

### Immunohistochemistry

MCA205 and MC38 tumors were fixed in 4% formaldehyde using Sarstedt formalin system overnight at 4 °C. Samples were transferred to 70% ethanol and stored at 4 °C until paraffin embedding. Tissues were embedded in paraffin and cut into 4 μm tissue sections. Hematoxylin and eosin staining was performed according to standard protocols. For CD34 immunostaining, 10 mM citrate buffer, pH 6.0 antigen retrieval, and rat anti-mouse CD34 (1:400, MEC 14.7, Novus Biologicals) were used. For CD8 immunostaining, proteinase K buffer antigen retrieval, rat anti-mouse CD8a (1:50, 4SM16, Invitrogen) were used. For Ki67 immunostaining, Target Retrieval Solution, Citrate pH 6.1 (DAKO), rabbit anti-mouse Ki67 (1:100, SP6, Abcam) were used. The sections were then stained with polyclonal rabbit anti-rat IgG (1:200, Dako), or EnVision rabbit (Dako) with NOVAred substrate (Vector). The tissue sections were counterstained with hematoxylin. The percentage of CD8 + or Ki67+ cells were quantified with Qupath software [51], using positive cell detection with optimized parameters. The percentage of CD34 + areas were quantified with Qupath software (ver. 0.2.3), using trained positive pixel classifier.

### Statistical analyses

Data analyses and graph generations were performed with Prism 6 (GraphPad) unless otherwise stated. Statistical analyses were performed using multiple *t* test with Bonferroni-Dunn correction for multiple comparisons. Tumor growth related statistics were analyzed with the open-access web tool TumGrowth (kroemerlab.shinyapps.io/TumGrowth/) [52]. Default settings were used.

## Supplementary information

Supplementary Figure S1–5

Supplementary Table 1. Antibodies used for flow cytometry analyses

Supplementary Table 2. Primer sequences for qRT-PCR on tumor or cell line RNA

Supplementary Table 3. Top 10 most upregulated and down regulated genes

Supplementary Table 4. List of differentially regulated genes
