# Supplementary material for: Blockade of beta-adrenergic receptors reduces cancer growth and enhances the response to anti-CTLA4 therapy by modulating the tumor microenvironment
Source: Oncogene. 2022 Jan 11;41(9):1364–75. doi: 10.1038/s41388-021-02170-0 (PMC8881216; doi:10.1038/s41388-021-02170-0)
Supplement: Supplementary file 1 — Supplementary Figure S1–5 [file 41388_2021_2170_MOESM1_ESM.pdf]

Supplementary figure S1

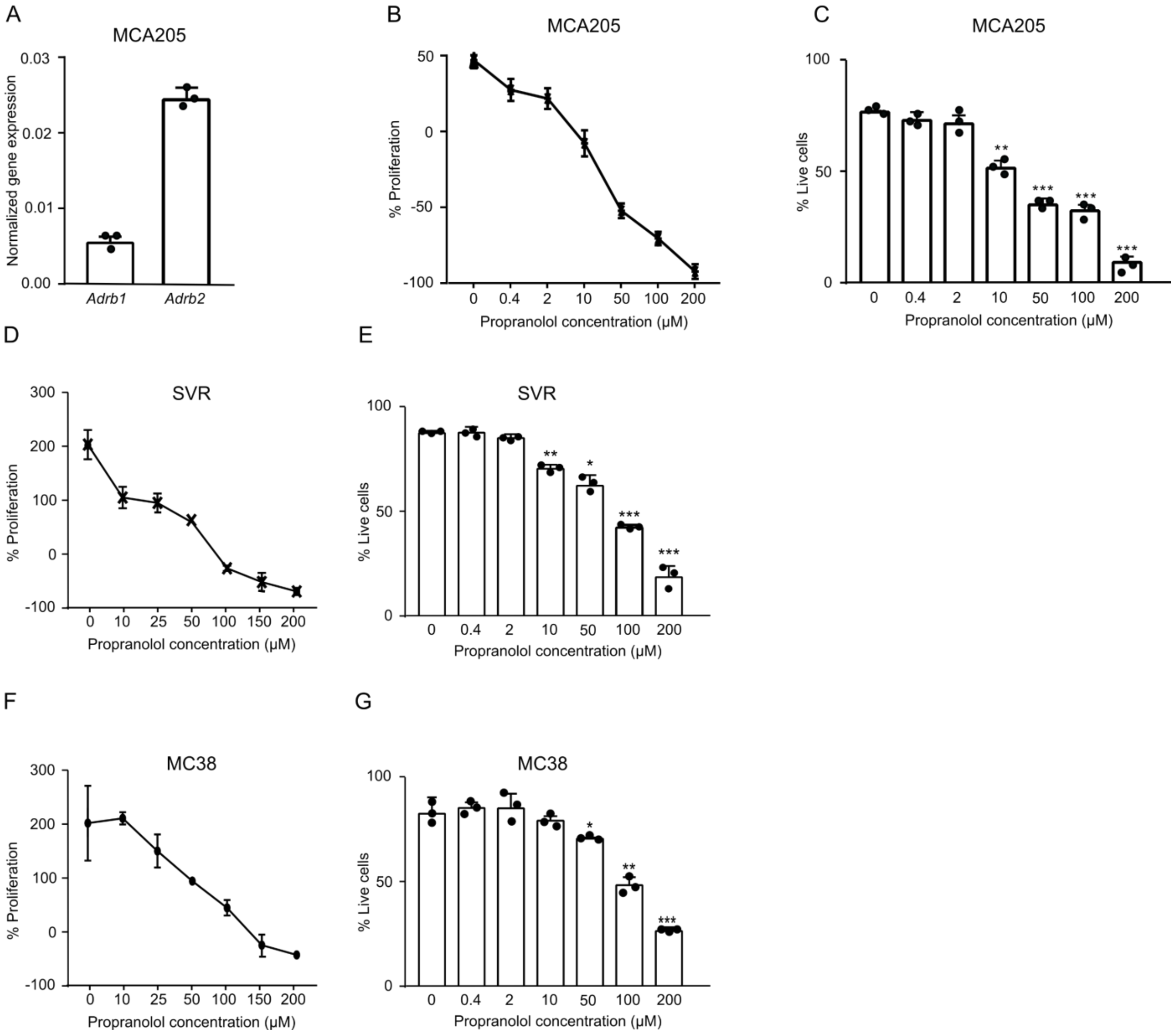

Figure S1. MCA205 fibrosarcoma, SVR angiosarcoma and MC38 carcinoma cells are sensitive to propranolol treatment. Dead cells were identified using trypan blue. Cell numbers are quantified manually using hemocytometer. **A,** Quantification of gene expression of *Adrb1*, *Adrb2* in in vitro cultured MCA205 cancer cells by qRT-PCR (normalized to Actb) (n=3, with 3 technical replicates each). **B-G,** Proliferation and viability of MCA205 **(B and C)**, SVR **(D and E)** or MC38 cells **(F and G)** after 24 hours of propranolol treatment at various concentrations (n=3). *p<0.05, **p<0.01, ***p<0.005, according to multiple t test with Bonferroni correction. Mean ± SD are depicted.

Supplementary figure S2

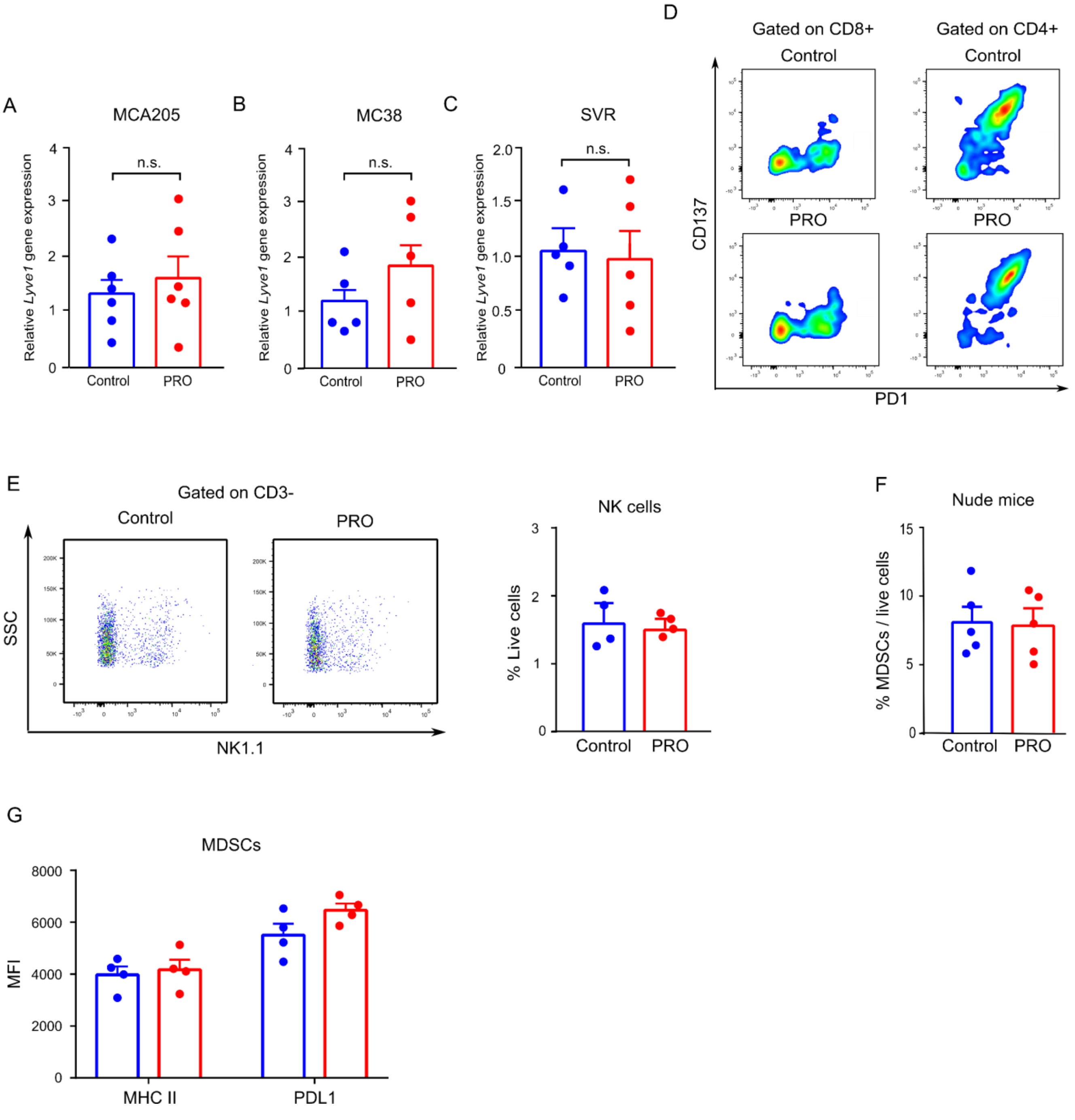

Figure S2. Effect of propranolol on lymphangiogenesis and immune cell composition in the TME. **A-C**, Quantification of lymphatic vessel marker *Lyve1* gene expression in MCA205 (**A**), MC38 (**B**), and SVR (**C**) tumors from control mice and propranolol (PRO) treated mice by qRT-PCR (n=5). **D-E,** Single cell suspensions were made from excised MCA205 tumors in C57BL/6 mice at the experimental endpoint and analyzed by flow cytometry (n=4). **D,** Representative flow cytometry dot plot (left) of PD1 and CD137 expression on tumor infiltrating CD4+ or CD8+ T cells. **E,** Representative flow cytometry dot plot (left) and quantification (right) of NK cells in the TME. Single cell suspensions were made from excised MCA205 tumors in nude mice at endpoint and analyzed by flow cytometry (n=5). **F,** Quantification of intratumoral MDSCs (CD11b+ F4/80- GR1+) in MCA205 tumors grown in nude mice. **G,** Quantifications of mean fluorescence intensity (MFI) of MHC II and PDL1 on intratumoral MDSCs in immune competent mice. Multiple t tests with Bonferroni correction for multiple comparison were used. n.s. not significant. Mean ± SEM are depicted.

Supplementary figure S3

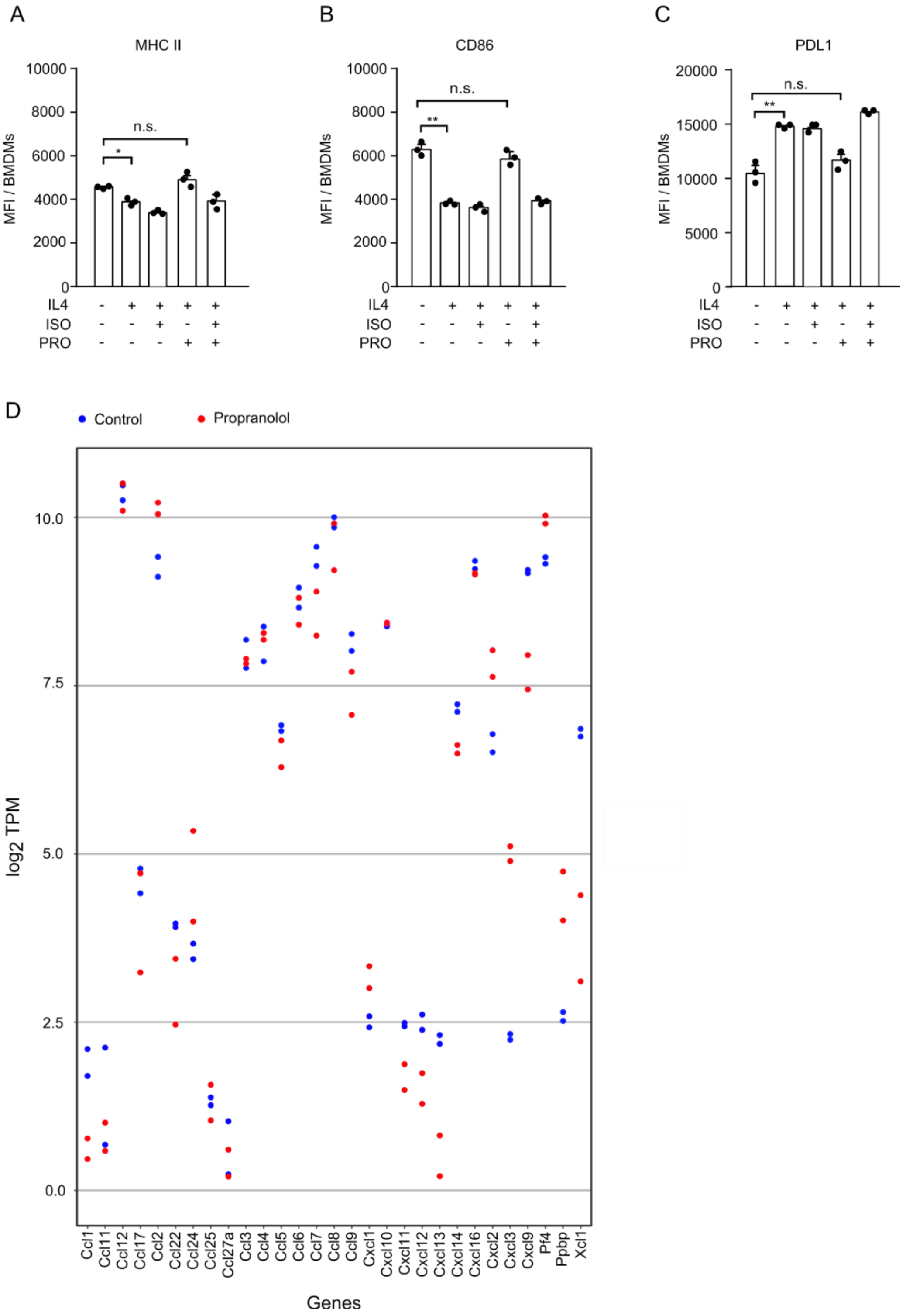

Figure S3. . Effect of propranolol on BMDMs and TAMs. **A-C,** BMDMs were stimulated with isoprenaline (ISO) or propranolol (PRO), in the presence of IL4, and cell surface marker expressions were evaluated by flow cytometry. Quantification of MHC II (**A**), CD86 (**B**), PDL1 (**C**) expression. **D,** TAMs from MCA205 tumors were sorted and subjected to RNA sequencing. **D,** The plot shows distinct chemokine expression in TAMs from the propranolol and control group. *p<0.05, **p<0.01, n.s. not significant, according to multiple t test with Bonferroni correction for multiple comparison. Mean ± SD are depicted.

Supplementary figure S4

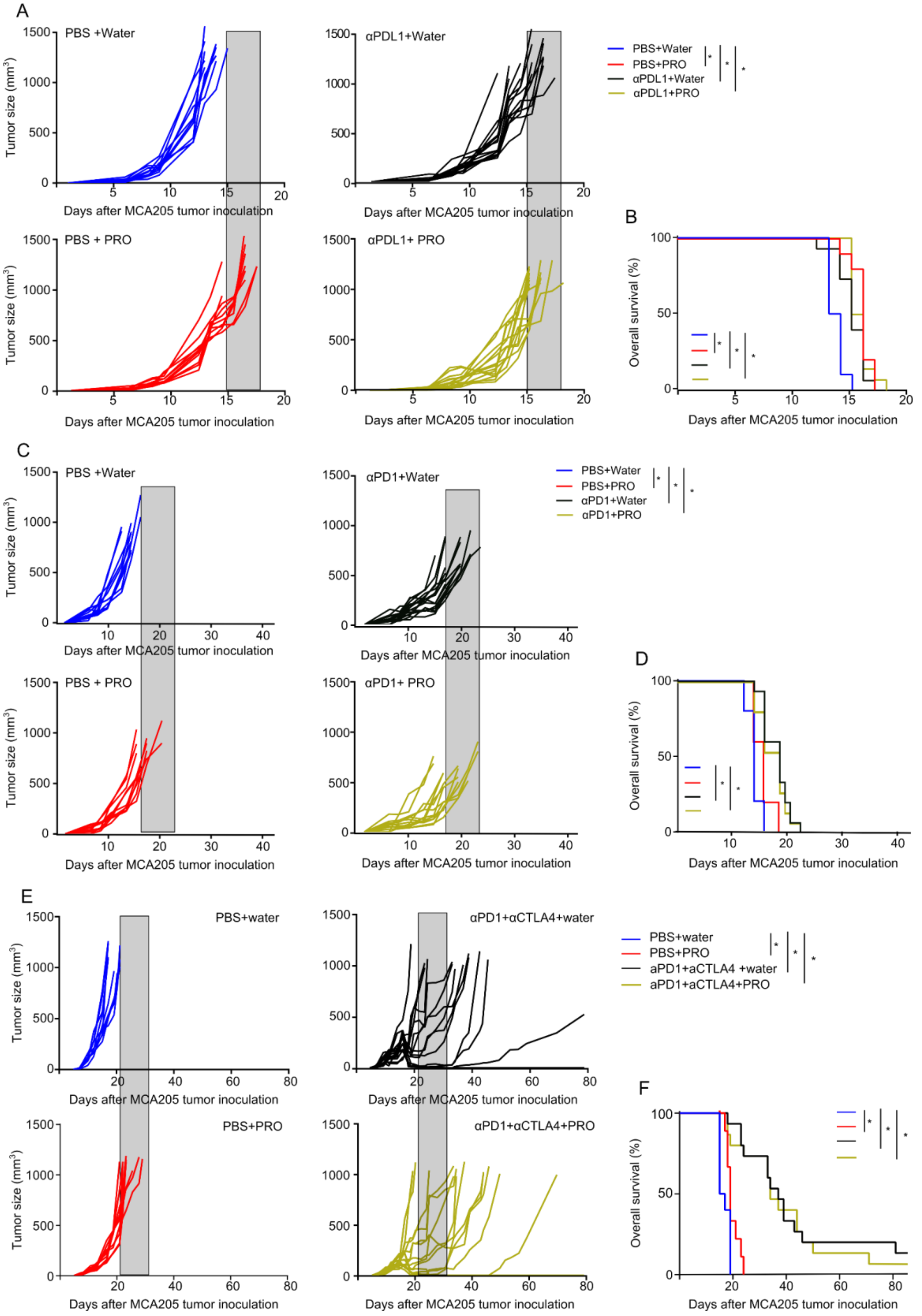

Figure S4. ADRB blockade by propranolol (PRO) does not improve the treatment efficacy of anti-PDL1, anti-PD1, and anti-PD1+anti-CTLA4 treatment in the MCA205 tumor model. C57BL/6 mice were inoculated with 10^6 MCA205 cells. **A&B,** Tumor growth kinetics (**A**), and Kaplan-Meier survival curves (**B**) of mice treated with anti-PDL1 and PRO. **C&D,** Tumor growth kinetics (**C**), and Kaplan-Meier survival curves (**D**) mice treated with anti-PD1, and PRO. **E&F,** Tumor growth kinetics (**E**), and Kaplan-Meier survival curves (**F**) of mice treated with anti-PD1, anti-CTLA4 and PRO. Each line represents one animal (n=10-15 per group). Shaded area represents the terminal tumor growth time frame of PBS+water group. Statistical analyses were performed using TumGrowth software. *p<0.05

Supplementary figure S5

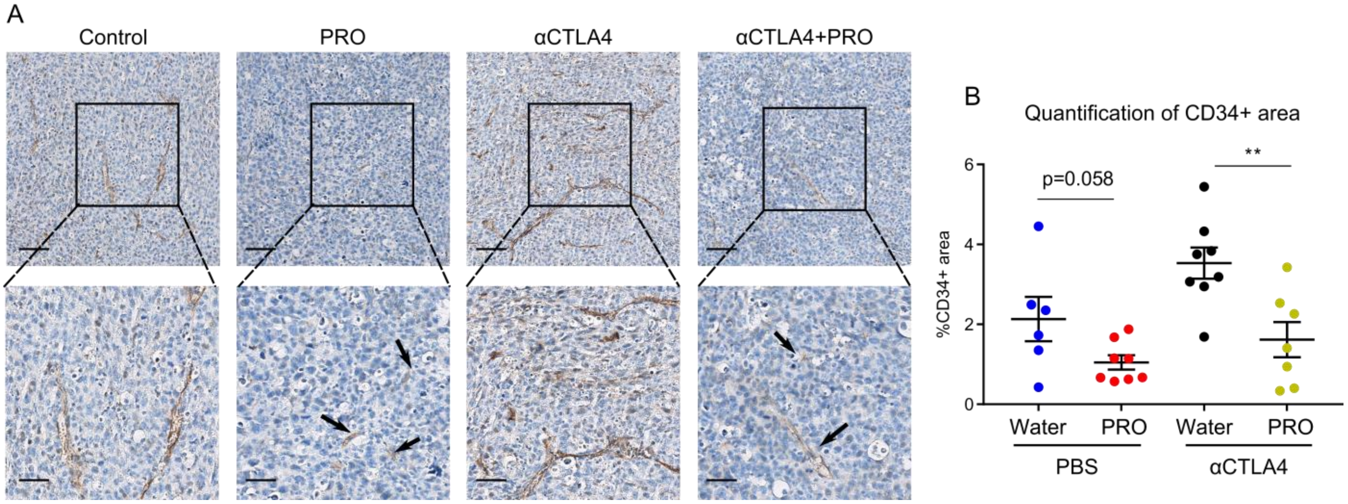

Figure S5. Propranolol reduces tumor angiogenesis in MC38 model. **A**. Representative CD34 (brown) IHC staining of MC38 tissues; hematoxylin counterstain; scale bar 100µm (upper panels), and 50µm (lower panels). **B**, Dot plot showing quantification of CD34 staining by percentages of DAB+ area. **p<0.01, according to multiple t test with Bonferroni correction. Mean ± SEM are depicted.
